# Comparison of intrafascial and non-intrafascial radical prostatectomy for low risk localized prostate cancer

**DOI:** 10.1038/s41598-017-17929-3

**Published:** 2017-12-14

**Authors:** Zhankui Zhao, Haizhou Zhu, Honglian Yu, Qingsheng Kong, Chengjuan Fan, Lin Meng, Chuanxin Liu, Xiegang Ding

**Affiliations:** 1 Department of urology, Affiliated hospital of Jining Medical University, Jining, Shandong 272100 P.R. China; 2 Department of Surgery Pandect, Clinical medical school, Jining Medical University, Jining, 272067 P.R. China; 3 Department of biochemistry, Basic medical school, Jining Medical University, Jining, 272067 P.R. China; 4 Collaborative Innovation Center, Jining Medical University, Jining, 272067 P.R. China; 5 Department of Urology, Zhongnan Hospital, Wuhan University, Wuhan, 430071 P.R. China

## Abstract

In this meta-analysis study, we compared the oncological and functional outcomes of intrafascial radical prostatectomy (IFRP) with non-intrafascial radical prostatectomy (NIFRP) in the treatment of patients with low risk localized prostate cancer (PCa). Relevant articles were identified by searching PubMed, EMBASE, Cochrane Library, Ovid, and the ISI Web of Knowledge databases. A total of 2096 patients were included from 7 eligible studies. Results of the pooled data showed that the oncological outcomes including gleason score, positive surgical margin and biochemical free survival rates were similar between the two groups. IFRP was superior to NIFRP with lower postoperative complication rates (RR 0.57, 95% CI 0.38, 0.85, p = 0.006), higher continence rates at 3 months post-operation (RR: 1.14; 95% CI, 1.04, 1.26; p = 0.006), and higher potency rates at 6 months (RR: 1.53; 95% CI, 1.07, 2.18; p = 0.02) and 12 months post-operation (RR: 1.38; 95% CI, 1.11, 1.73; p = 0.005). Additionally, there was a tendency towards higher potency rate in patients ≤65 years old compared with patients >65 years old after IFRP. Overall, these findings suggest that IFRP in young patients with low risk localized PCa had less postoperative complications, shortened time to return to continence and improved potency rate without compromising complete tumor control.

## Introduction

Prostate cancer (PCa) is the second most frequently diagnosed cancer in men worldwide^1^. Radical prostatectomy has undergone advances during the past 100 years. Walsh distinguished the locations of neurovascular bundles (NVBs) in relation to the fascial planes around the prostate, and hence the modified surgical techniques became possible to preserve the NVBs^2–4^. The NVBs were not just confined to a single bundle, but consist of variable distribution nerves in ventrolateral and dorsal position of periprostatic fascia^5–8^. Stolzenburg *et al*.^9^ reported that the intrafascial nerve sparing technique could preserve the nerve fibers surrounding the fascia of the prostate, which played a significant role in the preservation of potency and continence.

Improved potency and continence rates after intrafascial nerve sparing prostatectomy have been reported in many studies^10–12^. However, the controversial viewpoint deemed that the intrafascial technique might result in an incomplete resection and increased the rate of positive surgical margin (PSM), which correlated with the biochemical free survival (BFS) rate closely^13–15^. Attaining the best possible outcome in potency and continence might compromise the complete tumor control. Thus, the technique of intrafascial nerve sparing prostatectomy still needs to be evaluated in the treatment of localized PCa. Recently, several studies comparing intrafascial radical prostatectomy (IFRP) and non-intrafascial (interfascial, extrafascial and no nerve-sparing) radical prostatectomy (NIFRP) for low risk localized PCa have been reported in various medical centers from different countries^16–22^, but the oncological and functional results differed. So, this systematic review and meta-analysis was conducted based on the current available evidences.

## Results

### Search Results

**Figure 1:**
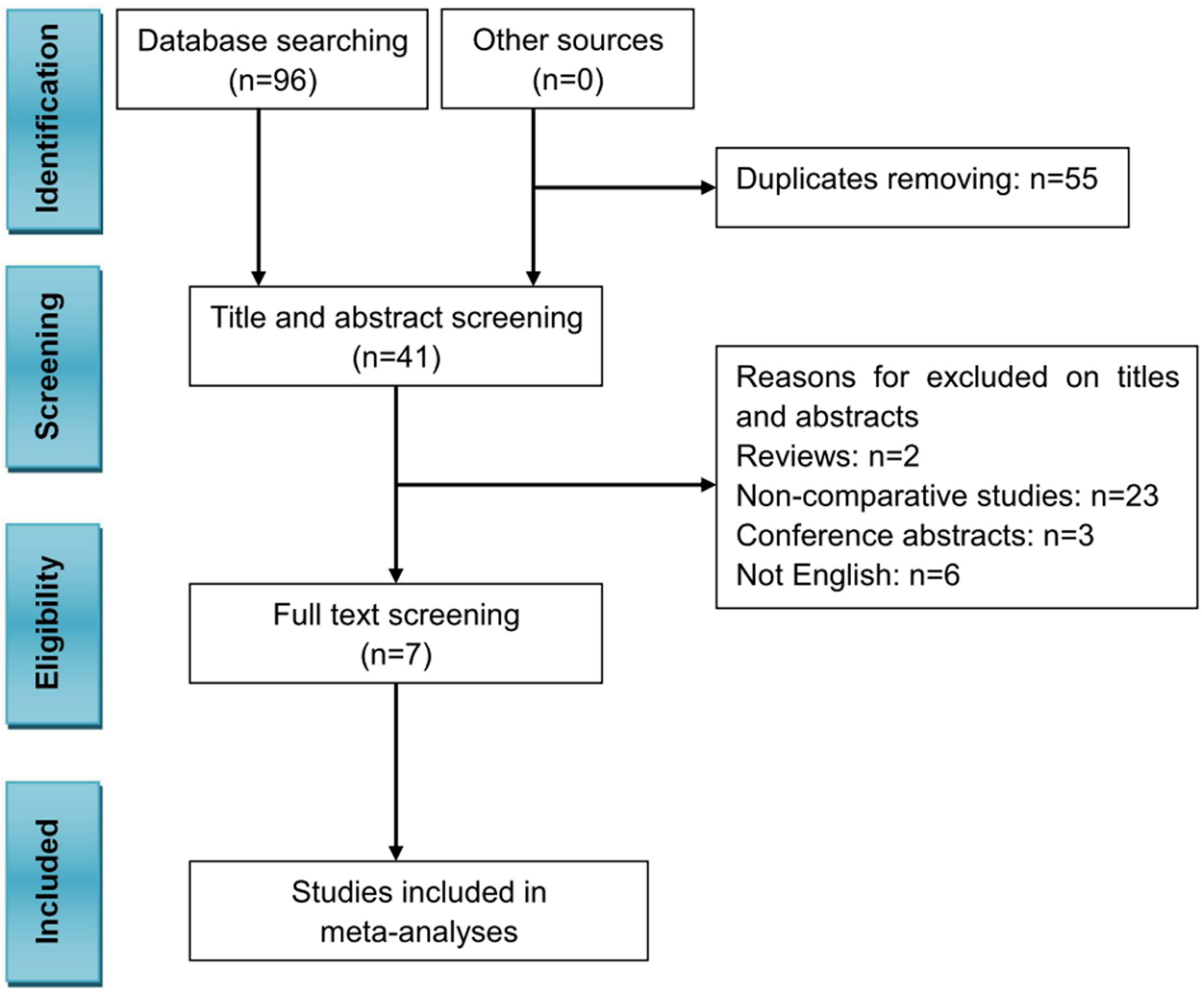
PRISMA flowchart of literature searches and results. PRISMA: Preferred Reporting Items for Systematic Reviews and Meta-analyses, RCTs: randomized controlled trials, NRCCTs: non-randomized concurrent controlled trials.

The initial search obtained 96 articles. After removing duplicates, there were 41 articles. After screening the abstracts and the full texts, 7 articles were selected for this study based on our eligibility criteria, which included 1 randomized controlled trail (RCT) and 6 non-randomized concurrent controlled trials (NRCCTs). A detailed Preferred Reporting Items for Systematic Reviews and Meta-analyses (PRISMA) flowchart (Fig. 1) was presented for the selection process.

### Characteristics of included studies

Major characteristics of the included studies were shown in Table 1. Of the 7 studies included, 2 were performed in the UK (Mischel 2009, Grant 2011), 1 in the USA (Lindsay 2009), 1 in Greece (Jens-Uwe 2010) and the remaining studies (Ashkan 2012, Tao 2013, Wael 2014) in Switzerland, China, and Germany, respectively, during the period between 2009 and 2014. The 7 studies included a total of 2096 patients who underwent radical prostatectomy for prostate cancer. Of them, 998 (47.6%) patients were treated with IFRP and 1098 (52.4%) patients were treated with NIFRP. From the preoperative oncological characteristics of the included studies, there was a visualized decreased trend of prostate specific antigen (PSA) level and Gleason score in IFRP group compared to NIFRP group.

**Table 1:** Characteristics of the studies included in the meta-analysis.

First author/year	Institution	Intervention	Patients	Age (Mean)	BMI (Mean)	PSA (Mean)	Biopsy Gleason score (Mean)	Oncological outcome	Functional outcome
Lindsay 2009	Robert Wood Johnson Medical School, New Brunswick, New Jersey	IFRP	70	58.71	NA	6.19	6.32	1, 3, 4	I, II
		NIFRP	77	58.56	NA	5.76	6.49		
Mischel 2009	Royal Surrey County Hospital, Guildford, Surrey, UK	IFRP	240	59	26	6.6	6.1	1, 4, 5	I, II
		NIFRP	270	59	26	6.5	6		
Jens-Uwe 2010	University of Patras Medical School, Patras, Greece	IFRP	200	60	NA	6.0	NA	1, 2, 3, 4, 5	I, II
		NIFRP	200	62	NA	6.8	NA		
Grant 2011	University of Edinburgh, Edinburgh, UK	IFRP	102	61.5	NA	4.9	6	1, 2, 3, 4, 5	I, II
		NIFRP	126	63.5	NA	7.3	7		
Ashkan 2012	University Hospital Zürich, Zürich, Switzerland	IFRP	80	62	25.57	7.16	NA	1, 2, 3, 4	NA
		NIFRP	106	64	26.44	11.53	NA		
Tao2013	Military Postgraduate Medical College, Beijing, China	IFRP	65	65	25.92	5.12	6	3, 4, 5	I, II
		NIFRP	130	65	25.89	5.98	6		
Wael2014	Ludwig-Maximilians-University Munich, Munich, Germany	IFRP	241	63.4	25.5	5.6	NA	1, 2, 3, 4, 5	I, II
		NIFRP	189	63.4	25.9	7.0	NA		

Oncological outcome: 1, complication rate 2.Gleason score 3, pT stage 4, positive surgical margin 5, biochemical free survival; Functional outcome: I, continence II, potency; IFRP: intrafascial radical prostatectomy, NIFRP: non-intrafascial radical prostatectomy, BMI: Body mass index; NA: not available.

Surgical technique details used in the 7 studies were shown in Supplementary Table S1. Among them, 4 studies compared intrafascial approach with interfascial approach. One study (Grant 2011) compared intrafascial approach with non-nerve sparing approach. One study (Ashkan 2012) compared the intrafascial approach with interfascial, extrafascial and no nerve-sparing approaches. One study (Mischel 2009) compared the intrafascial approach with standard nerve-preserving approach. Two studies (Lindsay 2009, Ashkan 2012) used robot-assisted laparoscopic devices. Four studies (Mischel 2009, Jens-Uwe 2010, Grant 2011, Tao 2013) used traditional laparoscopic devices. One study (Wael 2014) used open retropubic approach. Four studies (Lindsay 2009, Ashkan 2012, Tao 2013, Wael 2014) applied the athermal technique and two studies (Tao 2013, Wael 2014) preserved dorsal venous complex during IFRP.

The functional outcome (continence and potency) evaluation factors of the 7 studies were shown in Supplementary Table S2. Continence was evaluated by pads usage. Three studies (Lindsay 2009, Mischel 2009, Wael 2014) defined continence by requiring 0 pads per day and three studies (Jens-Uwe 2010, Grant 2011, Tao 2013) defined continence by requiring 0–1 safety pads per day. Requirement for 2–3 pads daily in patients during normal physical activity was considered as “mild incontinence” and more than 3 pads daily as “moderate and severe incontinence”. Potency was evaluated by various questionnaires, such as the expanded prostate cancer index composite (EPIC), International Index of Erectile Function (IIEF), Sexual Health Inventory for Men (SHIM), Sexual Encounter Profile (SEP), and erection was satisfactory for intercourse (ESI). Three studies (Mischel 2009, Jens-Uwe 2010, Wael 2014) used IIEF questionnaire, one study (Lindsay 2009) used EPIC questionnaire, one study (Tao 2013) used SHIM questionnaire, and one study (Grant 2011) used ESI questionnaire. Although the above questionnaires differed, they are still considered reliable for potency evaluation. Overall, all the questionnaires included two critical aspects, erectile function and intercourse satisfaction.

Table 2 presented the methodological quality of 7 studies. According to the Jadad scale^23^ for RCTs, one study (Jens-Uwe 2010) scored 3 points. According to the Newcastle-Ottawa Scale^24^, two NRCCT studies (Lindsay 2009, Grant 2011) scored 5 points and the other four studies (Mischel 2009, Ashkan 2012, Tao 2013, Wael 2014) scored 6 points.

**Table 2:** Quality assessment of the studies included in the meta-analysis.

First author/year	Study type	Quarlity assessment scale	Quarlity score
Lindsay 2009	NRCCT	Newcastle-Ottawa Scale	5 out of 9 points
Mischel 2009	NRCCT	Newcastle-Ottawa Scale	6 out of 9 points
Jens-Uwe 2010	RCT	Jadad scale	3 out of 5 points
Grant 2011	NRCCT	Newcastle-Ottawa Scale	5 out of 9 points
Ashkan 2012	NRCCT	Newcastle-Ottawa Scale	6 out of 9 points
Tao 2013	NRCCT	Newcastle-Ottawa Scale	6 out of 9 points
Wael 2014	NRCCT	Newcastle-Ottawa Scale	6 out of 9 points

RCT: randomized controlled trial, NRCCT: non-randomized concurrent controlled trials.

### Oncological outcomes

#### Complication rate

**Figure 2:**
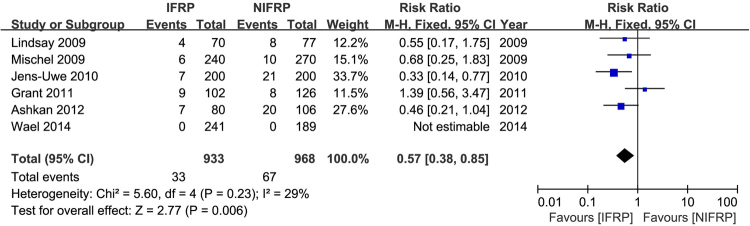
Forest plot and meta-analysis of complication rate. IFRP: intrafascial radical prostatectomy; NIFRP: non-intrafascial radical prostatectomy.

Six studies reported complication rates in 1901 included patients. The pooled data showed a higher complication rate in patients undergoing NIFRP (fixed-effect model, RR: 0.57, 95% CI 0.38, 0.85, p = 0.006) (Fig. 2).

#### Surgical specimen Gleason score

Three studies reported surgical specimen Gleason score in 794 included patients. In the subgroup analysis, the pooled data showed no significant difference between IFRP and NIFRP groups at Gleason score 4–6 (random-effect model, RR: 1.91; 95% CI, 0.87, 4.19; p = 0.11), Gleason score 7 (random-effect model, RR: 0.70; 95% CI, 0.42, 1.15; p = 0.16), and Gleason score 8–10 (random-effect model, RR: 0.53; 95% CI, 0.19, 1.47; p = 0.22) (Supplementary Fig. S1).

#### Surgical specimen pT stage

**Figure 3:**
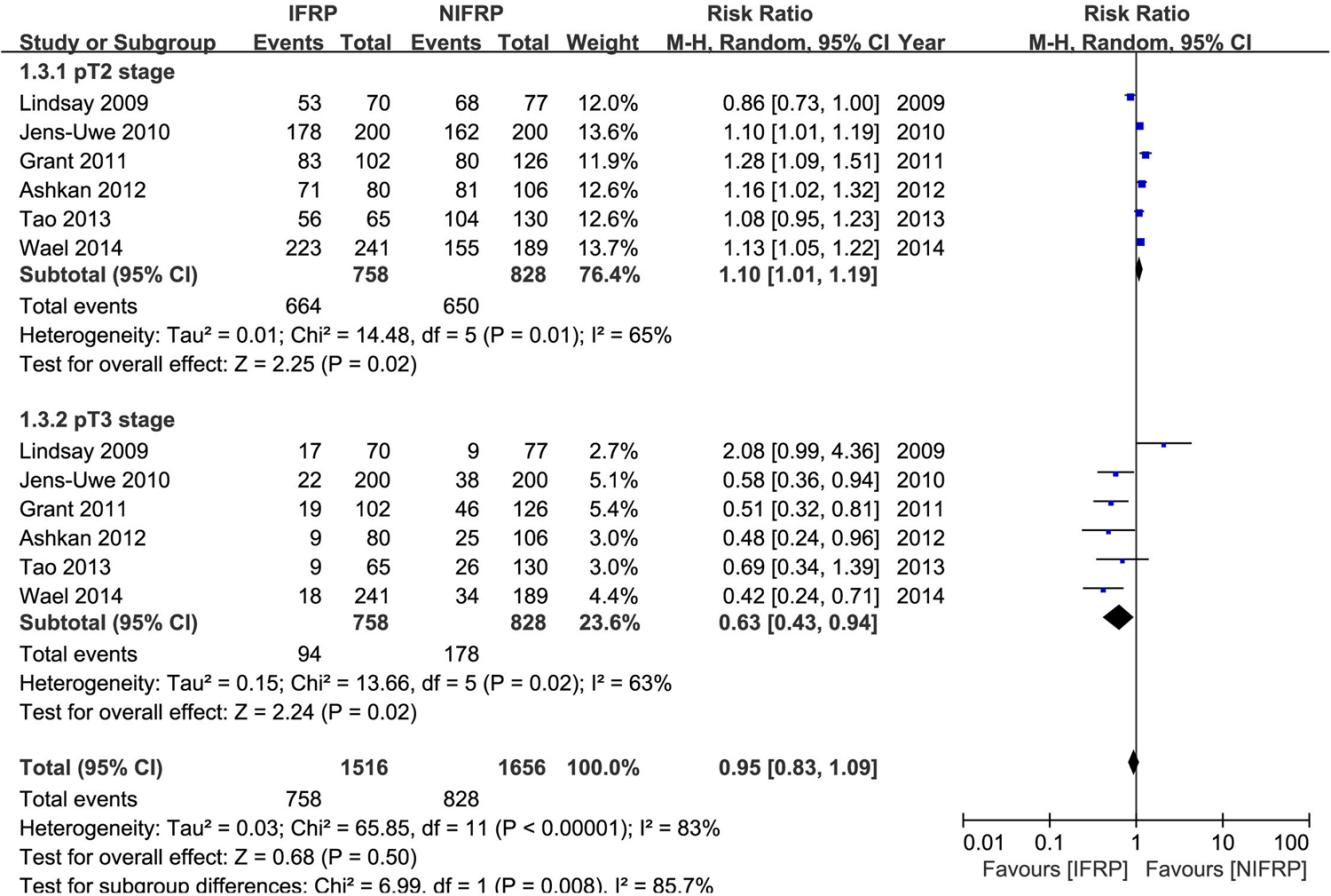
Forest plot and meta-analysis of surgical specimen pT stage. IFRP: intrafascial radical prostatectomy; NIFRP: non-intrafascial radical prostatectomy.

Figure 3 presented the surgical specimen pT stage in 1586 included patients. In the subgroup analysis, the pooled data showed a significantly higher rate in IFRP at pT2 stage (random-effect model, RR: 1.10; 95% CI, 1.01, 1.19; p = 0.02). But there was a significantly higher rate in patients who underwent NIFRP at pT3 stage (random-effect model, RR: 0.63; 95% CI, 0.43, 0.94; p = 0.02).

#### PSM rate

**Figure 4:**
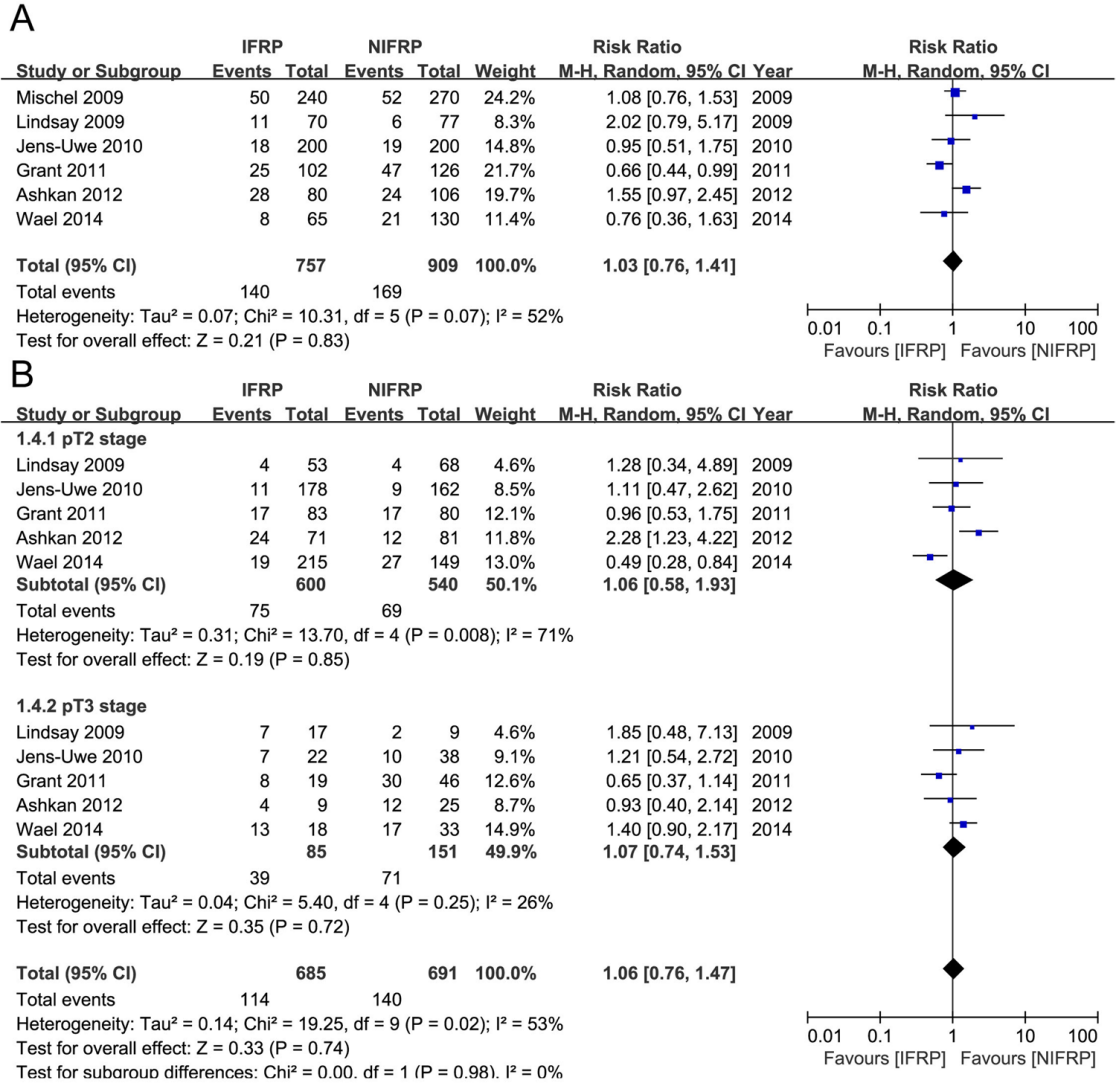
Forest plot and meta-analysis of PSM rate. (**A**) PSM rate at total pT stages; (**B**) PSM rate at different pT stages. PSM: positive surgical margin, IFRP: intrafascial radical prostatectomy, NIFRP: non-intrafascial radical prostatectomy.

Figure 4 presented the PSM rate in the included studies. Three studies compared the PSM rate of all pT stages in 1666 patients. Pooled data showed no significant differences between IFRP and NIFRP groups in total pT stages (random-effect model, RR: 1.03; 95% CI, 0.76, 1.35; p = 0.83). In the subgroup analysis, no significant difference was observed between IFRP and NIFRP at pT2 stage (random-effect model, RR: 1.06; 95% CI, 0.58, 1.93; p = 0.85), and pT3 stage (random-effect model, RR: 1.07; 95% CI, 0.74, 1.53; p = 0.72). Supplementary Fig. S2 presented the PSM rate at different locations of the prostate. In the subgroup analysis, no significant difference was observed between IFRP and NIFRP at apical (fixed-effect model, RR: 1.08; 95% CI, 0.81, 1.43; p = 0.61), basal (fixed-effect model, RR: 0.75; 95% CI, 0.23, 2.43; p = 0.63), and radial (fixed-effect model, RR: 1.18; 95% CI, 0.78, 1.79; p = 0.42) locations of the prostate.

#### BFS rate

**Figure 5:**
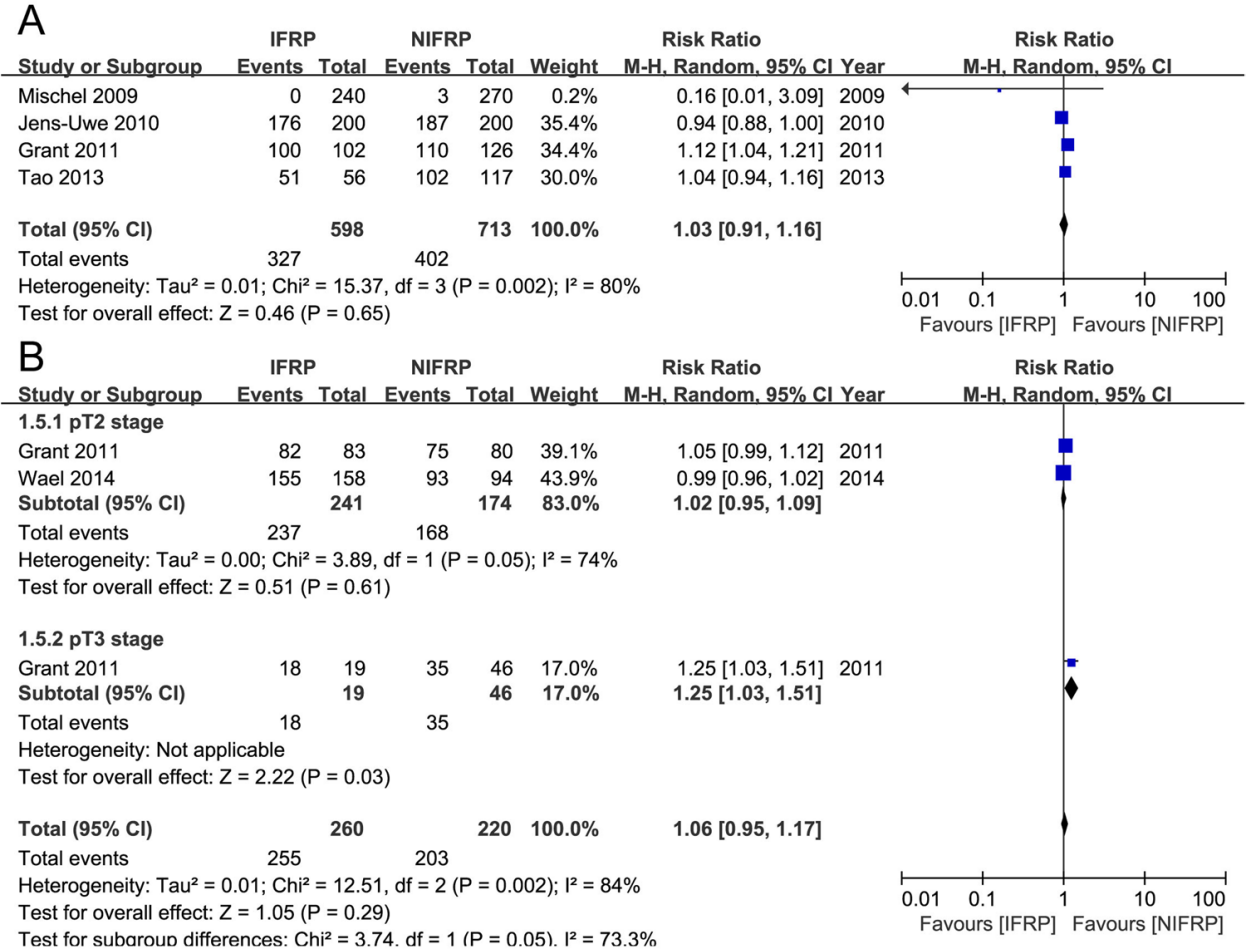
Forest plot and meta-analysis of BFS rate. (**A**) BFS rate at total pT stages; (**B**) BFS rate at different pT stages. BFS: biochemical free survival, IFRP: intrafascial radical prostatectomy, NIFRP: non-intrafascial radical prostatectomy.

Figure 5 presented the BFS rates at total and different pT stages. Three studies compared the BFS rate at total pT stages in 1311 patients, and reported no significant difference between IFRP and NIFRP (random-effect model, RR: 1.03; 95% CI, 0.91, 1.16; p = 0.65). In the subgroup analysis, two studies compared the BFS rate at pT2 stage in 415 patients, and showed no significant difference between IFRP and NIFRP (random-effect model, RR: 1.02; 95% CI, 0.95, 1.09; p = 0.61). One study compared the BFS rate at pT3 stage in 65 patients, and showed a significant higher BFS rate in IFRP group than NIFRP group (random-effect model, RR: 1.25; 95% CI, 1.03, 1.51; p = 0.03).

### Functional outcomes

#### Continence

**Figure 6:**
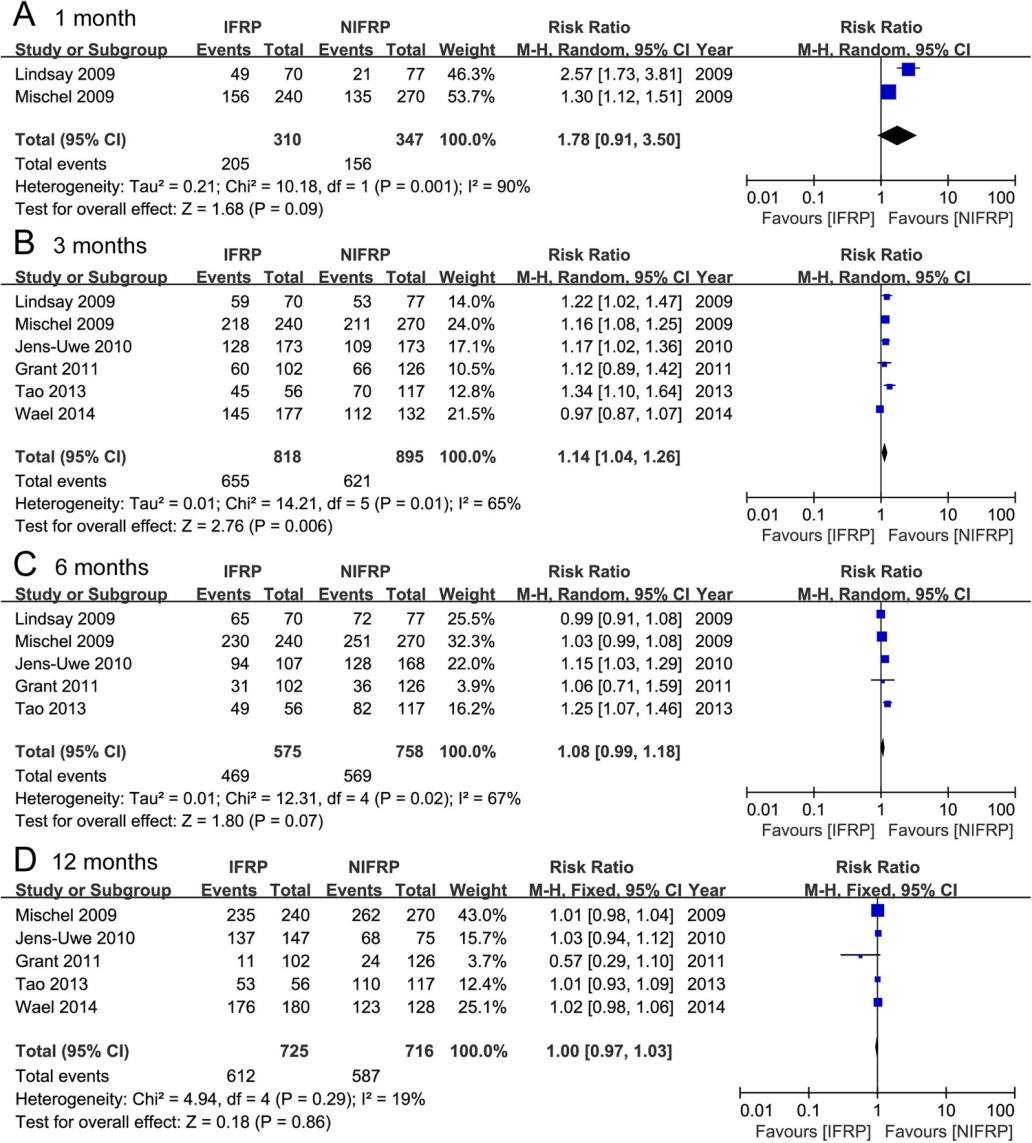
Forest plot and meta-analysis of continence rate at 1, 3, 6, and 12 months post-operation. IFRP: intrafascial radical prostatectomy, NIFRP: non-intrafascial radical prostatectomy.

Six studies analyzed the continence rate (0–1 pad usage) of 1713 included patients (Fig. 6). In the subgroup analysis, the pooled data showed a significantly higher continence rate in the IFRP group than NIFRP group at 3 months post-operation (random-effects model, RR: 1.14; 95% CI, 1.04, 1.26; p = 0.006). Additionally, a tendency towards higher continence rate was visualized in IFRP group at 1 month (random-effects model, RR: 1.78; 95% CI, 0.91, 3.50; p = 0.09) and 6 months post-operation (random-effects model, RR: 1.08; 95% CI, 0.99, 1.18; p = 0.07), although no statistical significance was observed. There was no significant difference between IFRP and NIFRP groups at 12 months post-operation (fixed-effects model, RR: 1.00; 95% CI, 0.97, 1.03; p = 0.86).

Four studies analyzed the mild incontinence rate (2–3 pads usage) in 747 included patients (Supplementary Fig. S3). In the subgroup analysis, the pooled data showed a significantly higher mild incontinence rate in NIFRP group than IFRP group at 6 months post-operation (fixed-effects model, RR: 0.62; 95% CI, 0.41, 0.93; p = 0.02). But there was no significant difference between IFRP and NIFRP groups at 3 months (fixed-effects model, RR: 0.92; 95% CI, 0.71, 1.32; p = 1.19) and 12 months post-operation (fixed-effects model, RR: 0.55; 95% CI, 0.25, 1.20; p = 0.13).

Three studies analyzed the moderate and severe incontinence rates (more than 3 pads usage) for 747 included patients (Supplementary Fig. S4). The pooled data showed a significantly higher moderate and severe incontinence rates in NIFRP group than IFRP group at 3 months (fixed-effects model, RR: 0.40; 95% CI, 0.25, 0.65; p = 0.0001), and 6 months post-operation (fixed-effects model, RR: 0.25; 95% CI, 0.10, 0.64; p = 0.004). No significant difference was observed at 12months post-operation (fixed-effect model, RR: 0.36; 95% CI, 0.13, 1.06; p = 0.06).

#### Potency rate

**Figure 7:**
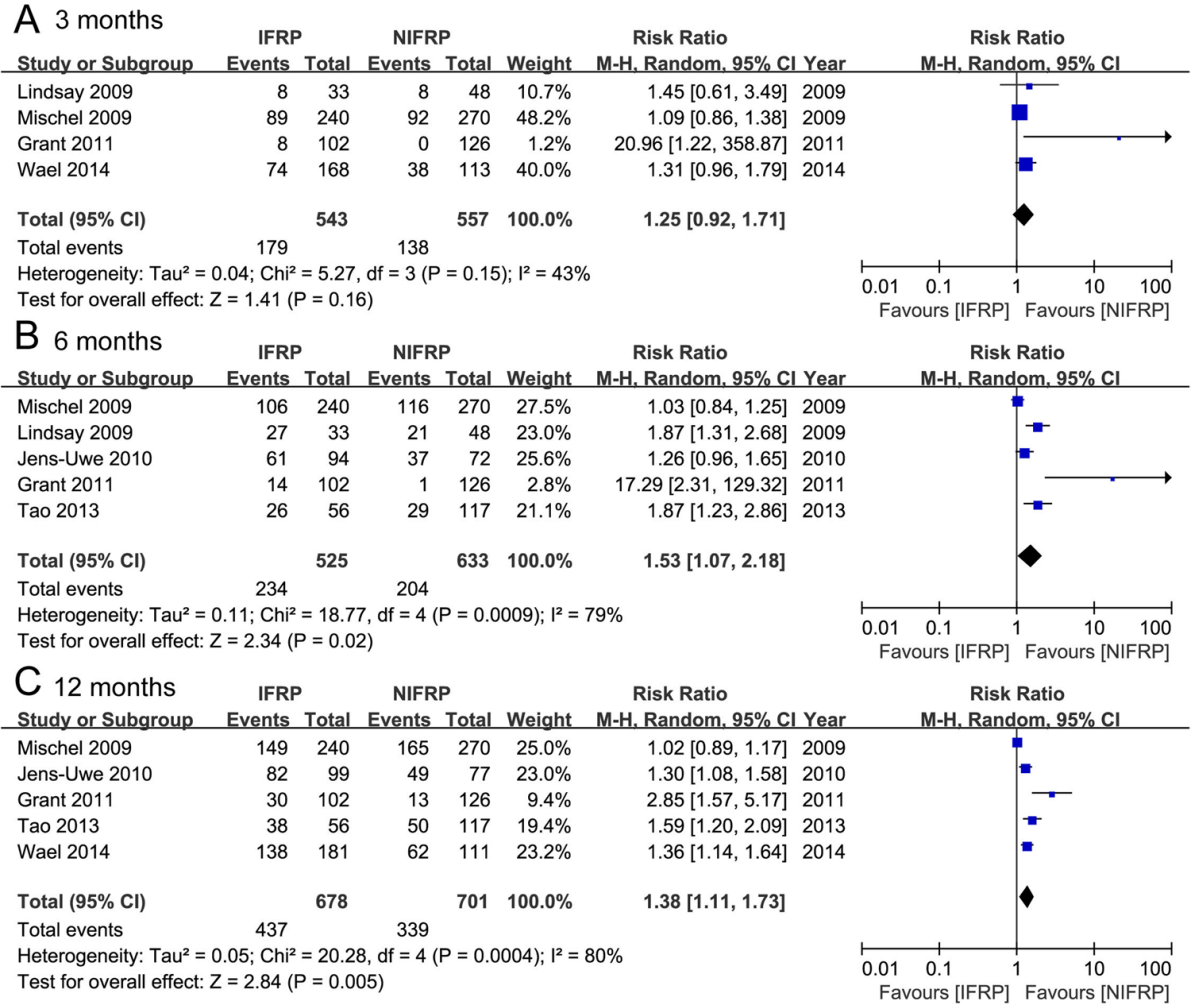
Forest plot and meta-analysis of potency rate at 3, 6, and 12 months post-operation. IFRP: intrafascial radical prostatectomy, NIFRP: non-intrafascial radical prostatectomy.

Figure 7 listed out the potency rates at different time points (3, 6, and 12 months). The pooled data showed a significantly higher potency rate in IFRP group than NIFRP group at 6 months (random-effects model, RR: 1.53; 95% CI, 1.07, 2.18; p = 0.02), and 12 months post-operation (random-effects model, RR: 1.38; 95% CI, 1.11, 1.73; p = 0.005), although no significant difference was observed at 3 months post-operation (random-effects model, RR: 1.25; 95% CI, 0.92, 1.71; p = 0.16).

In two studies, the patients in both the groups were divided into two subgroups by their age (greater or less than 65 years old). The potency rates of the above two subgroups at 6 and 12 months post-operation were analyzed. At 6 months post-operation, the pooled data showed a significantly higher potency rate in IFRP group than NIFRP group in patients ≤65 years old (fixed-effects model, RR: 1.44; 95% CI, 1.12, 1.84; p = 0.004). However, no significant difference was observed in patients >65 years old (fixed-effects model, RR: 1.64; 95% CI, 0.95, 2.80; p = 0.07), (Supplementary Fig. S5A). At 12 months post-operation, the pooled data showed a significantly higher potency rate in IFRP group than NIFRP group both in patients ≤65 years old (fixed-effects model, RR: 1.33; 95% CI, 1.14, 1.56; p = 0.004) and patients >65 years old (fixed-effects model, RR: 1.48; 95% CI, 1.01, 2.17; p = 0.04) (Supplementary Fig. S5B). Additionally, the pooled data indicated a tendency towards higher potency rate in patients ≤ 65 years old compared with patients >65 years old after IFRP.

Grant D. Stewart *et al*.^19^ divided the patients into two groups (greater or less than 60 years old) and analyzed the potency rate. Results showed that the potency rates of patients ≤60 years old were superior than patients >60 years old for both IFRP and NIFRP groups, but this a reached statistical significance at 6 months post-operation for IFRP group and at 12 months post-operation for NIFRP group. In men <60 years old undergoing IFRP, in whom recovery of erectile function was particularly important, 75% men had satisfied erection for intercourse at 12 months post-operation.

### Sensitivity analysis and publication bias

Sensitivity analysis was conducted by sequentially excluding each study, and the results remained statistically significant. This suggested that the results of this meta-analysis were stable. Supplementary Fig. S6 showed funnel plot of the studies included in this meta-analysis that reported complication rates. All studies lie inside the 95% CIs, with an even distribution around the vertical, indicating no obvious publication bias.

## Discussion

Low risk localized PCa has been defined as clinical stage T1-T2, with a biopsy Gleason score ≤6, and PSA <10 ng/ml. The perfect surgical technique for patients with low risk localized PCa is to eradicate the tumor while preserving sexual and urinary quality of life. Based on the updated anatomical and pathological studies, the IFRP nerve sparing technique was proposed to preserve the periprostatic fascia and NVBs by dissecting even closer to the prostate. This in turn results in maximizing the improvement of postoperative continence and potency rate without compromising the oncological efficacy^25–28^. Controversially, the IFRP nerve sparing technique might result in the increased rate of PSM and high biochemical recurrence risk^20,29^. This is the first systematic review and meta-analysis of all the currently available evidences regarding IFRP comparison with NIFRP. Overall, this meta-analysis compared the oncological and functional outcomes of IFRP and NIFRP for patients with low risk localized PCa. Results demonstrated that IFRP was safe, and significantly reduced the incontinence rate and maximized the preservation of potency.

For the oncological outcomes, five postoperative factors were compared. Our review showed no significant differences in the Gleason score, PSM and BFS. Nevertheless, we found a decreased tendency of complication rates with IFRP group. This might due to that the surgical trauma was less during the IFRP compared with NIFRP. The intrafascial procedure eliminated the incision of endopelvic fascia, mobilized the levator ani muscles, and even preserved the pubovesical complex in some medical centers^30,31^. The other reason for the low complication rates is that IFRP has a longer learning curve than NIFRP. Surgeons of the included studies who can perform IFRP usually experienced NIFRP primarily^16,19^. Hence, the complication rate of IFRP would be lower than NIFRP.

In our meta-analysis, the rate of surgical specimen pT stage was significantly different between the two groups. Three studies demonstrated a higher rate of pT2 stage in IFRP group, but a higher rate of pT3 stage in NIFRP group. The reason for this could be attributed to an earlier preoperative clinical stage, lower PSA level and Gleason score selected for the IFRP patients in some studies^18,20,22^. Our meta-analysis study demonstrated a significantly higher BFS rate in IFRP group than NIFRP group at pT3 stage. Because the periprostatic fascia involved in tumors were in T3 stage pathologically. There is a consensus of opinion that intrafascial tumor growth was contraindicated for IFRP^18,32^. So, the patient selection should be carefully done to eliminate the patients at T3 stage for IFRP. Currently, multiparametric Magnetic Resonance Imaging (mpMRI) was applied for PCa detection. MpMRI guided prostate biopsy and MRI-transrectal ultrasound fusion targeted biopsy improved the diagnostic accuracy of PCa^33–35^. So, we suggested that the patients selected for IFRP should receive mpMRI in the future studies.

PSM has been proposed as an independent predictor of PCa recurrence after radical prostatectomy, and was associated with an increased hazard of biochemical failure^14,15^. During IFRP, the dissection of the prostate was performed via prostatic fascia, and no periprostatic tissue was left overlying the prostate. Ashkan Mortezavi *et al*.^20^ reported that IFRP lead to a higher incidence of PSM. Wael Y Khoder *et al*.^22^ reported that IFRP less likely lead to PSM in pT2 stage, but associated with higher rates of PSM in pT3 stage. In our meta-analysis, the PSM rate was similar between the two groups. The PSM rate variations might be due to different pathological analysis techniques in different medical centers and the policy of routinely obtaining intraoperative frozen tissue sections. Histologically, pT3 stage PCa is diagnosed only in the presence of attached periprostatic fascia to the prostate specimen. This diagnosis is not theoretically possible because the periprostatic fascia was preserved during IFRP. Therefore, the intraoperative frozen tissue sections should be recommended routinely during IFRP. A positive frozen section is a clear indication to change IFRP to NIFRP during the operation. Moreover, Fromont *et al*.^36^ suggested that intraoperative frozen tissue section analysis is a reliable method to monitor the nerve sparing during prostatectomy. Nevertheless, the preoperative surgical indication for IFRP also should be confined strictly. Jeongyun Jeong *et al*.^13^ applied the Partin nomogram to select patients for nerving sparing surgery, and this method led to a significant decrease in the rate of PSM. Hence, Partin nomogram can be recommended as a supplement to patient selection criteria for IFRP.

For the functional outcomes, IFRP greatly improved potency rates and shortened the time to return to continence in our meta-analysis. Potency was defined according to the validated questionnaires in our included studies. There was a significantly higher potency rate in IFRP group than NIFRP group at 6 and 12 months post-operation, especially for patients <65 years old. During IFRP, as many nerve fibers as possible were preserved by periprostatic fascia preservation^37^. Graefen *et al*.^4^ demonstrated correlation between an increased number of preserved nerve fibers and improved postoperative potency. Kaiho *et al*.^38^ revealed that stimulation of ventrolateral periprostatic fascia induced an increase in the intracavernosal pressure by electrophysiological tests. Besides, another important technique to improve the postoperative potency is the energy-free approach during IFRP. Ahlering *et al*.^39^ reported that the avoidance of electrocautery near the NVBs afforded a five-fold increase in early return to potency. In our meta-analysis, four studies applied athermal technique to minimize the electrical current trauma to NVBs. Furthermore, the preoperative potency status and expectation of postoperative sexual function also should be considered before the selection of surgical methods. Young patients with low risk PCa and normal preoperative potency can be recommended to receive IFRP preferentially.

This meta-analysis showed that IFRP was associated with significantly improved continence rate at 3 months post-operation. This IFRP may indicate an advantage of the procedure in early postoperative continence. The preserved periurethral structures during IFRP, including endopelvic fascia, levator ani muscles and the puboprostatic ligament play an important role in the early recovery of continence^40^. Although no statistical significance regarding the continence was observed between the two groups at 6 and 12 months post-operation, there was a significantly higher incontinence rate (mild, moderate and severe) in NIFRP group than IFRP group at 6 months post-operation. These results demonstrated superiority of IFRP on continence recovery compared with NIFRP. However, the evaluation criteria for continence were according to the pad usage only in the included studies. The supplemental pad-weighting test should be recommended to validate the current results in the future studies^41^.

Several limitations in the present meta-analysis must be taken into account. Firstly, the main limitation was that the study included only one RCT, while the other studies were NRCCTs, including observational studies. Inadequate random sequence generation, allocation concealment, and blinding tended to increase the risk of bias. Results showed that IFRP was suitable for low risk PCa patients. But the patients in some observational studies were in the intermediate risk group with mean biopsy gleason score 7 (Grant 2011) or PSA >10ng/ml (Ashkan 2012), which in turn increased the risk of bias. In future, high-quality RCTs should be well-designed, randomized and include strictly selected patients. Secondly, heterogeneity still existed in our meta-analysis, although subgroup and sensitivity analysis were conducted to minimize the heterogeneity. Thirdly, the surgical performances in the seven studies were carried out by different levels of surgeons. The differences in the surgeons experience could influence the intraoperative and postoperative outcomes. In the NIFRP group, interfacial, extrafascial, and non-nerve sparing approaches were included. The different surgical approaches may also increase the risk of bias. The baseline information of patients’ erectile function and continence was not available from the 7 included studies, which is likely a source of bias. Fourthly, the sample size of this meta-analysis was small. Large sample size may help to obtain more enough statistical power for detecting the real results. Finally, the duration of follow-up period was short. Long-term outcomes, especially for BFS and the relationship between PSM and BFS, are needed to prove the results. Nevertheless, our meta-analysis study tried to fill the gap in the current literature on functional and oncological outcome comparisons between IFRP and NIFRP groups in PCa patients, providing the most up to date information in this area. Moreover, enough data had accumulated to allow an assessment based on meta-analytical methods.

## Methods

### Search Strategy

A literature search was performed in February 2017 using PubMed, EMBASE, Cochrane Library, Ovid, and ISI Web of Knowledge databases to identify relevant studies, without restriction to publication types or regions. The following medical subject heading (MeSH) terms and their combinations were applied to search the articles: prostate cancer, prostatectomy, intrafascial, interfascial, extrafascial and nerve-sparing. The function of related articles was also used to identify relevant manuscripts. References were explored to broaden the search strategy. The published language was restricted to English.

### Study selection

The study selection process was based on the PRISMA guidelines (www. prisma-statement.org) in a flow diagram. The included studies must meet the following criteria: (1) type of research: RCTs or NRCCTs; (2) compare intrafascial and non-intrafascial (interfascial, extrafascial and no nerve-sparing) techniques; (3) outcomes: including oncological outcomes (complication rate, gleason score, pT stage, PSM rate, BFS rate) and functional outcomes (continence and potency). The studies were excluded if: (1) the surgery was not radical prostatectomy; (2) intrafascial technique was not mentioned; (3) the studies did not report the detailed information of oncological or functional outcomes. Two authors (H.Y. and H.Z.) independently evaluated the eligibility of all the retrieved articles by inclusion and exclusion criteria. Disagreements were resolved by discussion or in consultation with an adjudicating senior author (Q.K.).

### Data extraction

Two of the authors (Z.Z. and H.Z.) reviewed the included studies and extracted data from each eligible study. Oncological outcomes between intrafascial and non-intrafascial surgical interventions were compared including complication rate, gleason score, pT stage, PSM, and BFS. Biochemical recurrence was defined as PSA never reached nadir(<0.1 mg/l) or PSA ≥0.2 mg/l after nadir. Functional outcomes evaluated included continence and potency.

### Quality assessmen**t**

The methodological quality was assessed by using Jadad scale^23^ for RCTs (score ranging between 0 and 5, with 0–2 being low, 3–5 high), and the Newcastle-Ottawa Scale^24^ for NRCCTs, which consisted of three factors: patient selection, comparability of the study groups, and assessment of outcomes (score ranging between 0 and 9 with 0–2 being low, 3–5 moderate, and 6–9 high).

### Statistical Analysis

Meta-analyses were performed using RevMan software (version 5.2, the Cochrane Collaboration). The RR and relevant 95% CIs were used to compare dichotomous variables. Heterogeneity was quantified using I^2^ statistics, with low, moderate, and high to I^2^ values of 40%, 70%, and 100%, respectively. When the I^2^ value was 40% or lower, indicating no evidence of heterogeneity, we used the fixed-effects model; otherwise, the random-effects model was used. Detailed subgroup analyses were performed by stratifying Gleason score, pT stage, pads usage for continence, different time points to evaluate continence and potency, and different ages to evaluate potency. The influence of a single study on the overall risk estimate was investigated by sequentially removing each study to test the robustness of the main results. Visual inspection of the funnel plots of the outcomes was applied to evaluate potential publication bias.

## Conclusions

IFRP in low risk localized PCa demonstrated less postoperative complications, shortened time to return to full continence and improved potency without compromising the complete tumor control. Compared with NIFRP, IFRP had equivalent Gleason score, PSM and BFS rates. Nevertheless, despite our rigorous methodology, the inherent limitations of included studies prevented us from reaching definitive conclusions, and the role of IFRP technique still remains to be defined. The surgical indications also should be considered carefully. Our meta-analysis results deduced that IFRP would be a preferred approach for young patients with low risk PCa and high expectation of postoperative sexual function. Moreover, the intraoperative frozen tissue sections should be routinely carried out during IFRP to ensure negative surgical margin. Future well-designed, large-scale, randomized, long-term follow-up multi-center RCTs are awaited to confirm and update the findings of our meta-analysis.

## Electronic supplementary material

Supplementary information
