# Supplementary material for: Comparison of intrafascial and non-intrafascial radical prostatectomy for low risk localized prostate cancer
Source: Sci Rep. 2017 Dec 14;7:17604. doi: 10.1038/s41598-017-17929-3 (PMC5730549; doi:10.1038/s41598-017-17929-3)
Supplement: Supplementary file 1 — Supplementary information [file 41598_2017_17929_MOESM1_ESM.pdf]

Title page

Comparision of intrafascial and non-intrafascial radical prostatectomy for low risk localized prostate cancer

Zhankui Zhao[1+], Haizhou Zhu[2+], Honglian Yu[3,4*], Qingsheng Kong[3,4], Chengjuan Fan[1], Lin Meng[1], Chuanxin Liu[4], and Xiegang Ding[5]

[1]Department of urology, Affiliated hospital of Jining Medical University, Jining, Shandong 272100, P.R. China.

[2]Department of Surgery Pandect, Clinical medical school, Jining Medical University, Jining, 272067, P.R. China.

[3]Department of biochemistry, Basic medical school, Jining Medical University, Jining, 272067, P.R. China.

[4]Collaborative Innovation Center, Jining Medical University, Jining, 272067, P.R. China

[5]Department of Urology, Zhongnan Hospital, Wuhan University, Wuhan 430071, P.R. China

*Correspondence and requests for materials should be addressed to Honglian Yu (e-mail: yuhonglian@mail.jnmc.edu.cn)

[+]These authors contributed equally to this work.

Supplementary Tables

Supplementary Table S1. Surgical technique factors of the studies included in the meta-analysis

First author/year	Intervention	Single surgeon	Surgical approach	Nerve-sparing	Endopelvic fascia	Prostatic pedicle dissection	DVC control
Lindsay	IFRP	Yes	Robot-assisted laparoscopic 1ntrafascial	Bilateral	Preserved	Athermal	Ligated
2009	NIFRP	Yes	Robot-assisted laparoscopic interfascial	Bilateral	Incisied	Athermal	Ligated
Mischel	IFRP	Yes	Laparoscopic intrafascial	Bilateral	Preserved	UltraCision	Ligated
2009	NIFRP	Yes	Laparoscopic standard	Bilateral	Incisied	UltraCision	Ligated
Jens-Uwe	IFRP	No	Laparoscopic intrafascial	Bilateral	Preserved	UltraCision	Ligated
2010	NIFRP	No	Laparoscopic interfascial	Bilateral	Incisied	UltraCision	Ligated
Grant	IFRP	Yes	Laparoscopic intrafascial	Bilateral	Preserved	UltraCision	Ligated
2011	NIFRP	Yes	Laparoscopic non nerve sparing	Non	Incisied	UltraCision	Ligated
Ashkan	IFRP	No	Robotic-Assisted laparoscopic intrafascial	Bilateral	Preserved	Athermal	Ligated
2012	NIFRP	No	Robotic-Assisted laparoscopic inter-/extrafascial or no nerve-sparing	Unilateral/non	Incisied	Athermal /ultraCision	Ligated
Tao	IFRP	Yes	Laparoscopic intrafascial	Bilateral	Preserved	Athermal	Preserved
2013	NIFRP	Yes	Laparoscopic interfascial	Bilateral	Incisied	Athermal	Preserved
Wael	IFRP	No	Open; retropubic intrafascial	Bilateral	Preserved	Athermal	Preserved
2014	NIFRP	No	Open; retropubic interfascial	Bilateral	Incisied	Athermal	Preserved

IFRP: intrafascial radical prostatectomy, NIFRP: non-intrafascial radical prostatectomy, DVC: dorsal venous complex, NA: not available.

Supplementary Table S2. Functional outcome evaluation factors of the studies included in the meta-analysis

First author/year	Continence definition	Potency definition	Data collection	Follow up (moths)
Lindsay 2009	0 pad (EPIC questionnaire #5)	EPIC questionnaire #18, #19 and SHIM questionnaire	Validated questionnaire	9
Mischel 2009	0 pad	IIEF questionnaire	Validated questionnaire	12
Jens-Uwe 2010	0-1 safety pad (ICS questionnaire)	IIEF questionnaire, SEP questionnaire	Validated questionnaire	12
Grant 2011	0-1 safety pad	ESI	Interview	24
Ashkan 2012	NA	NA	NA	23
Tao 2013	0-1 safety pad (Modified symptom questionnaire)	SHIM questionnaire	Validated questionnaire	12
Wael 2014	0 pad	IIEF-5	Validated questionnaire	12

EPIC: expanded prostate cancer index composite, SHIM: Sexual Health Inventory for Men, IIEF: International index of erectile function, ICS: International Continence Society, SEP: Sexual Encounter Profile, ESI: Erection was satisfactory for intercourse, NA: not available.

Supplementary Figures

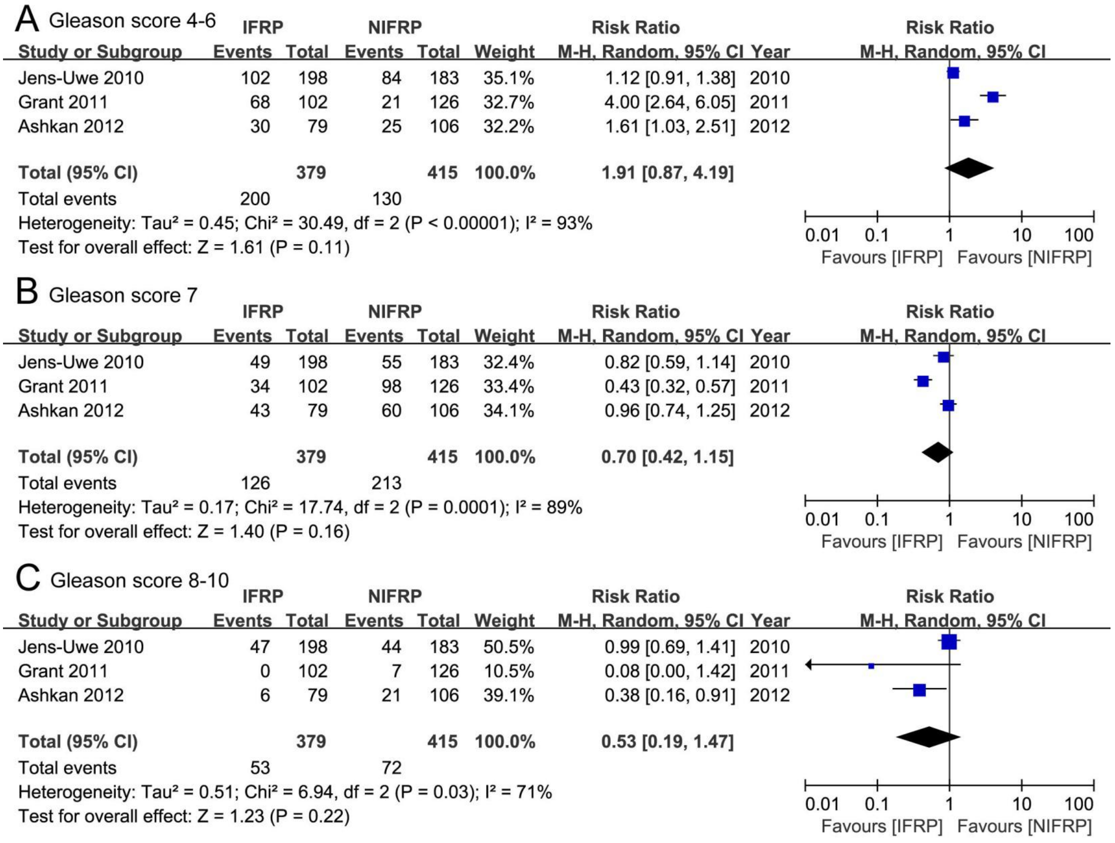

Supplementary Figure S1. Forest plot and meta-analysis of surgical specimen Gleason score. (**A**) Gleason score 4-6; (**B**) Gleason score 7; (**C**) Gleason score 8-10. IFRP: intrafascial radical prostatectomy; NIFRP: non-intrafascial radical prostatectomy.

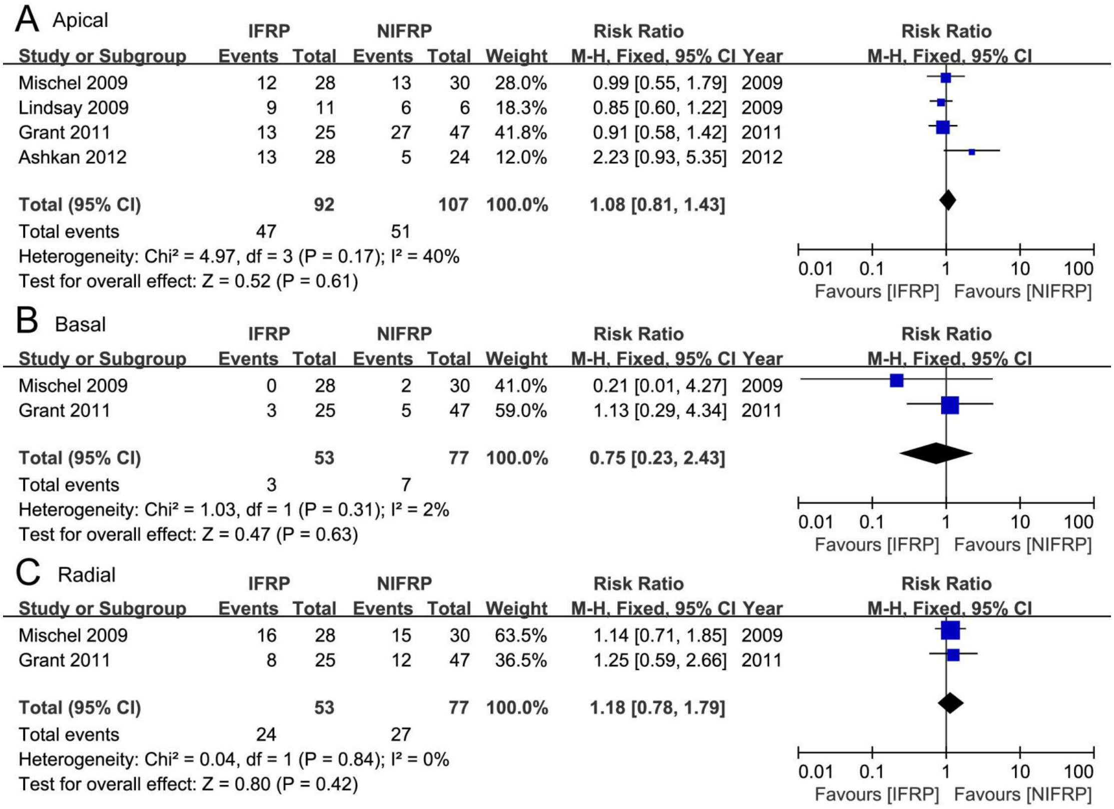

Supplementary Figure S2. Forest plot and meta-analysis of PSM rate at different locations of the prostate. (**A**) Apical; (**B**) Basal; (**C**) Radial. PSM: positive surgical margin, IFRP: intrafascial radical prostatectomy, NIFRP: non-intrafascial radical prostatectomy.

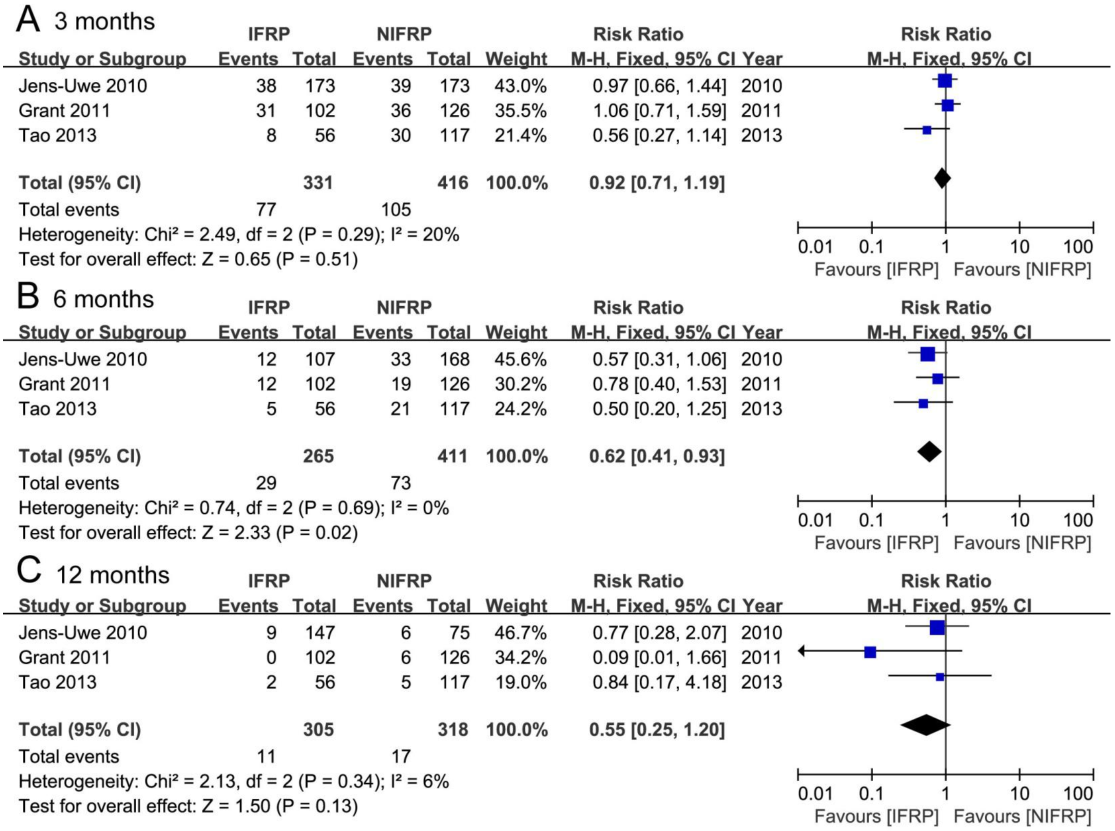

Supplementary Figure S3. Forest plot and meta-analysis of mild incontinence rate at 3, 6, and 12 months post-operation. IFRP: intrafascial radical prostatectomy, NIFRP: non-intrafascial radical prostatectomy.

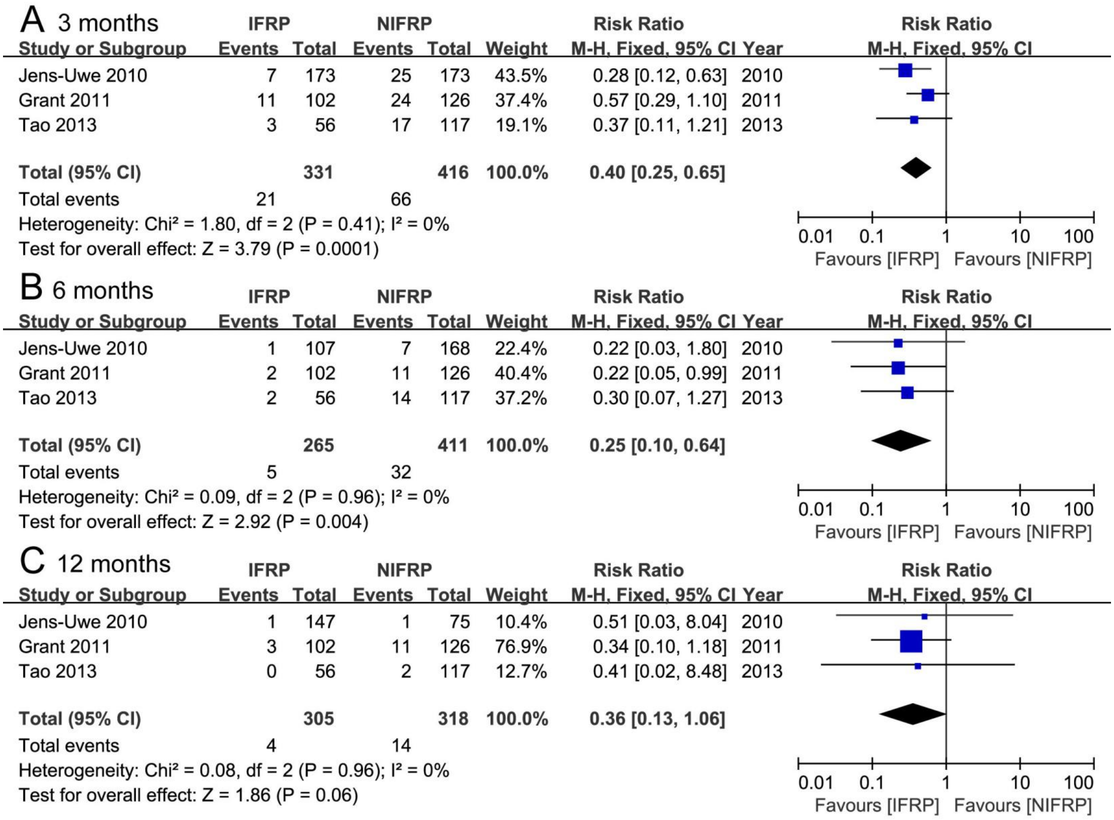

Supplementary Figure S4. Forest plot and meta-analysis of moderate and severe incontinence rate at 3, 6, and 12 months post-operation. IFRP: intrafascial radical prostatectomy, NIFRP: non-intrafascial radical prostatectomy.

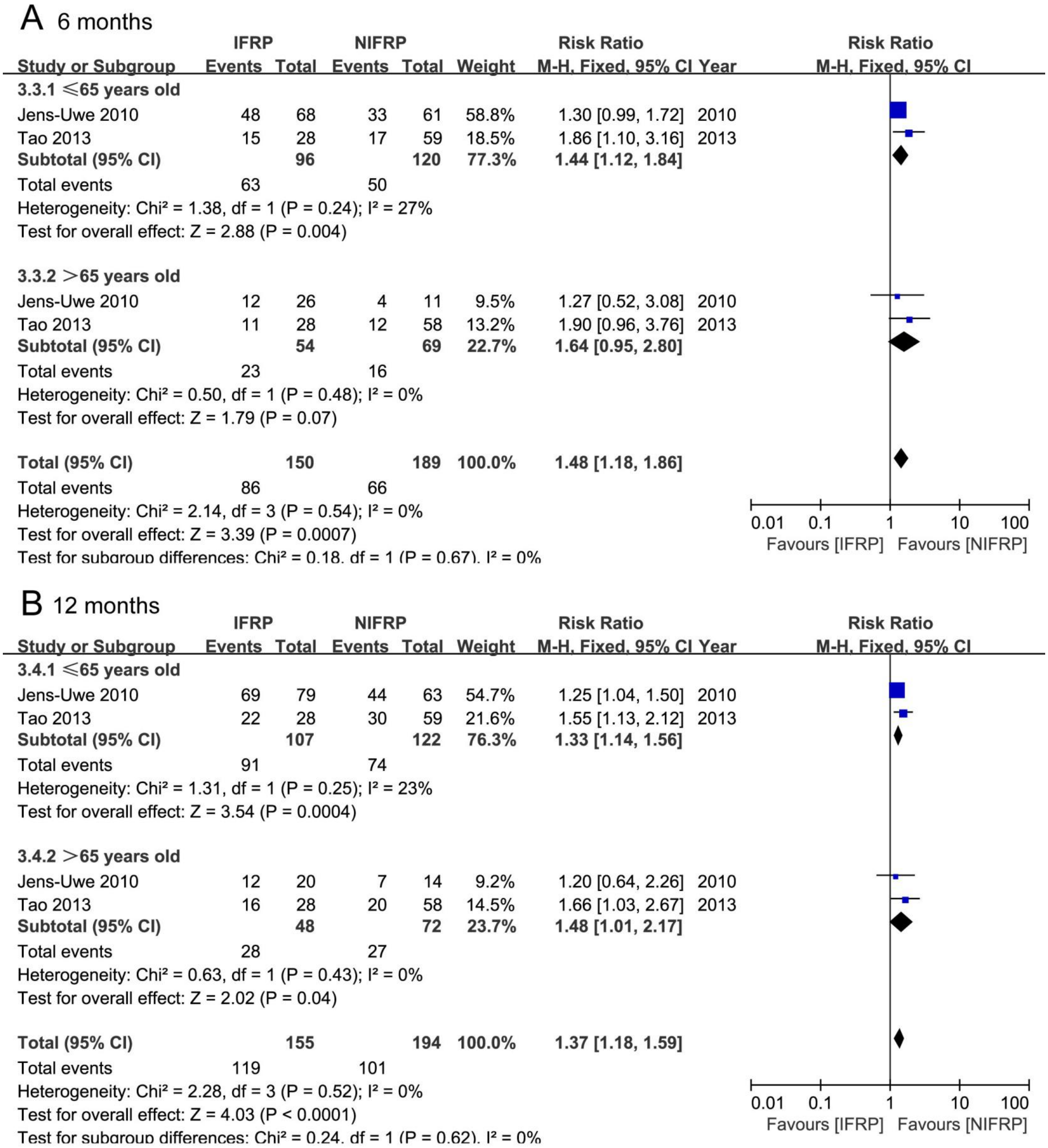

Supplementary Figure S5. Forest plot and meta-analysis of potency rate in subgroups (greater or less than 65 years old). (**A**) 6 months; (**B**) 12 months. IFRP: intrafascial radical prostatectomy, NIFRP: non-intrafascial radical prostatectomy.

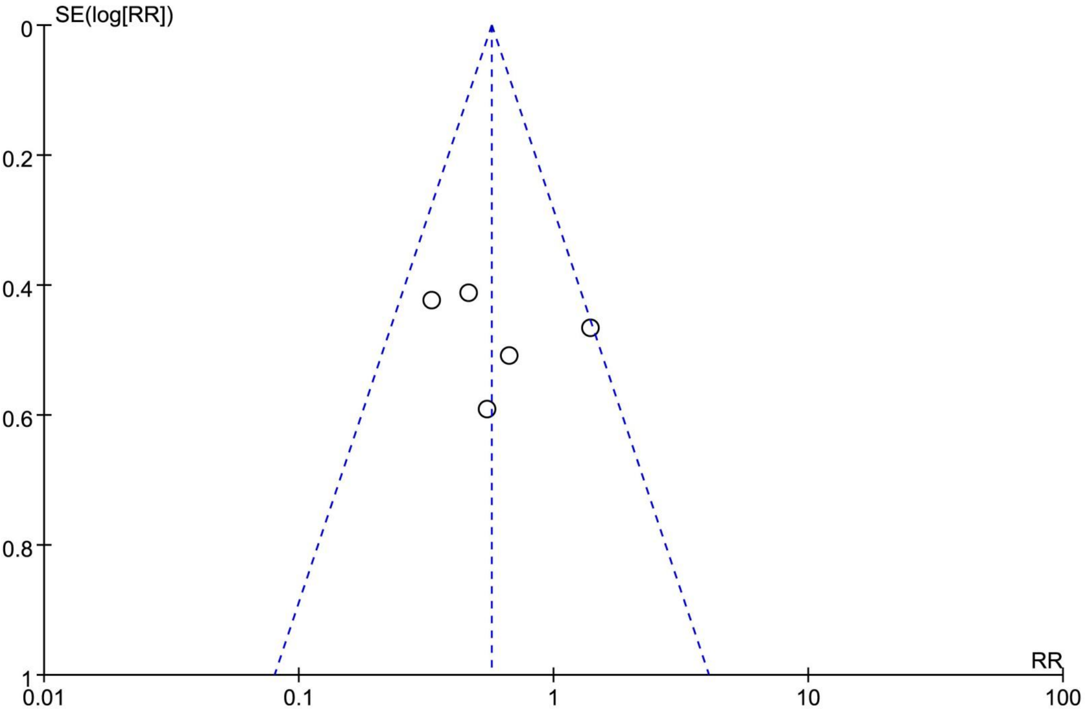

Supplementary Figure S6. Funnel plot illustrating meta-analysis of complication rate. SE: standard error, RR: risk ratio.
